# Turning Seasonal Signals into Segmentation Cues: Recolouring the Harmonic Normalized Difference Vegetation Index for Agricultural Field Delineation

**DOI:** 10.3390/s25185926

**Published:** 2025-09-22

**Authors:** Filip Papić, Luka Rumora, Damir Medak, Mario Miler

**Affiliations:** Faculty of Geodesy, University of Zagreb, Kačićeva 26, 10000 Zagreb, Croatia; filip.papic@geof.unizg.hr (F.P.); luka.rumora@geof.unizg.hr (L.R.); damir.medak@geof.hr (D.M.)

**Keywords:** field delineation, NDVI, harmonic analysis, cylindrical colour spaces, zero-shot segmentation, SAM, phenology

## Abstract

**Highlights:**

**What are the main findings?**

- Recolouring harmonic Normalized Difference Vegetation Index (NDVI) into cylindrical colour spaces enables zero-shot field delineation without retraining.
- The ordinal day harmonic fit separates parcels better than the annual radian fit on the tested area of interest.

**What is the implication of the main finding?**

- A simple, scalable, interpretable baseline is identified for operational parcel monitoring, with low engineering overhead, and no training datasets beyond evaluation.
- The baseline is easy to extend by adding Intersection-over-Union–Non-Maximum Suppression (IoU-NMS) or topology repair, and by swapping NDVI for other indices where seasonality is informative.

**Abstract:**

Accurate delineation of fields is difficult in fragmented landscapes where single-date images provide no seasonal cues and supervised models require labels. We propose a method that explicitly represents phenology to improve zero-shot delineation. Using 22 cloud-free PlanetScope scenes over a 5 × 5 km area, a single harmonic model is fitted to the NDVI per pixel to obtain the phase, amplitude and mean. These values are then mapped into cylindrical colour spaces (Hue–Saturation–Value, Hue–Whiteness–Blackness, Luminance-Chroma-Hue). The resulting recoloured composites are segmented using the Segment Anything Model (SAM), without fine-tuning. The results are evaluated object-wise, object-wise grouped by area size, and pixel-wise. Pixel-wise evaluation achieved up to F1 = 0.898, and a mean Intersection-over-Union (mIoU) of 0.815, while object-wise performance reached F1 = 0.610. HSV achieved the strongest area match, while HWB produced the fewest fragments. The ordinal time-of-day basis provided better parcel separability than the annual radian adjustment. The main errors were over-segmentation and fragmentation. As the parcel size increased, the IoU increased, but the precision decreased. It is concluded that recolouring using harmonic NDVI time series is a simple, scalable, and interpretable basis for field delineation that can be easily improved.

## 1. Introduction

The precise delineation of agricultural parcels is the basis for land management, subsidy control, precision agriculture, and compliance with environmental regulations [1,2]. This task is difficult in fragmented landscapes, where parcels vary in shape, size, farming practises, and phenology, and where fixed physical boundaries are not typical [3]. Conventional pipelines typically rely on high-resolution RGB or multispectral single-date images, which require significant manual effort to obtain accurate results and still lack important seasonal features [4,5]. Single-date inputs are either spectrally flat, as is the case with RGB images, or temporally blind, as is the case with a single multispectral scene, so they lack the phenological cues that are important for boundary delineation [6]. There are workarounds using deep learning, but they usually require large, annotated datasets and extensive training, which limits their use [7,8].

In the past, field delineation relied on manual digitisation or semi-automated aerial photography and cadastral methods, which were accurate but labour-intensive, time-consuming, and costly, especially in areas with highly fragmented parcels or rapid changes in land use [9]. The advent of satellite remote sensing has changed the data supply, and enables large-scale, timely, and cost-effective monitoring [10]. Modern delineation algorithms can be divided into three categories: edge detection, region-based, and integrated hybrid methods [11]. A recent comprehensive review of agricultural parcel and boundary delineation confirms that the overwhelming majority of methods rely on spatial or spectral features, with deep learning models dominating recent work, while temporal phenology is typically treated only as an auxiliary input [12]. This imbalance underscores that phenology remains underused in the context of field delineation, despite the increasing availability of dense satellite imagery time series.

Harmonic analysis provides a compact, interpretable summary of seasonal dynamics, and has proven useful in plant monitoring, land use mapping, and anomaly detection [13,14]. It decomposes the NDVI time series into mean, amplitude, and phase, and isolates cyclical vegetation dynamics from long-term trends, which are then used in the recolouring step. Modern zero-shot segmentation (e.g., the Segment Anything Model) can suggest segmentation masks without domain-specific training, but the effectiveness depends on how clearly the input expresses the boundary-relevant contrasts [15]. Cylindrical colour spaces (HSV, HWB, LCH) are a natural way to represent complementary cues as separable channels (timing, seasonal range, mean level) for both human and algorithmic interpretation [16]. A common challenge in remote sensing is persistent cloud cover and irregular acquisition, which can create gaps in the NDVI time series. Such missing data reduces the robustness of seasonal signal extraction. This limitation is increasingly mitigable. Harmonic regression, based on the Fourier series, has been shown to interpolate through noisy or incomplete records while preserving phenological dynamics, which makes it a promising tool for agricultural monitoring even under cloud-prone conditions [17,18]. Uniline linear interpolation or composing, harmonic regression yields smooth, periodic representations of vegetation dynamics, and is robust against residual atmospheric noise such as haze and cirrus clouds [19]. As such, studies [17,18] have demonstrated its effectiveness for filling temporal gaps in Landsat and Sentinel time series.

Amin et al. [20] propose an operational ResUNet-a d7 multi-task network using freely available Sentinel-2 monthly syntheses, coupled with a Gaussian Mixture post-processing step to refine adjacency boundaries. By assessing 14 sites in France over two years, their system attains a weighted F1 score of 92%, and it is explicitly designed for early-season delineation with single-date composites, which limits dependence on long, cloud-free time series and heavy preprocessing [20]. The study also documents practical constraints of their approach, and argues for methods that can operate with minimal temporal input, especially early in the season. Their observations motivated our choice to encode phenology compactly, via harmonic NDVI, and to keep the segmenter zero-shot and training free.

In parallel, recent works also target crop mapping, which is methodologically relevant because it demonstrates how temporal cues and ensembles improve agricultural inference at scale. Hosseini et al. [21] developed a stack ensemble on Google Earth Engine (GEE) that fuses Sentinel-2 and Landsat-8/9 monthly composites. Four base learners are combined using a Minimum-Distance meta-classifier, achieving an overall accuracy of 94.24% in 2021, and maintaining 90.97–91.82% overall accuracy when temporally transferred to other years without retraining [21]. While [21] focuses on classifying Cropping Intensity Patterns, not boundary extraction, their findings strengthen our premise that temporally informed representations are decisive in agricultural remote sensing.

Similarly, the authors of [22] present an ensemble-based framework for corn and soybean mapping on GEE. Their pipeline first injects the phenological priors by restricting analysis to phenophases with maximal separability, then augmenting the spectra with Gray-Level Co-occurrence Matrix (GLCM) texture features and performs feature-importance filtering before collective voting across three supervised classifiers [22]. The validation is performed at field scale in Illinois, U.S.A., and Indiana, U.S.A., and the study underscores three design lessons that translate to delineation. The first lesson is that phenology-aware timing increases class separability, the second one is that texture and spatial cues complement spectra when intra-class variability is high, and finally, simple ensembles can be robust and scalable in GEE [22]. Our method uses a compact temporal signal, and exposes it as perceptually separable channels, after which it leverages a zero-shot segmenter.

Amin et al. [20] demonstrate that strong geometry can be recovered without long time series when the signal is well structured, while ensemble crop-mapping efforts [21,22] show that phenology and aggregation across learners boost robustness and transferability at scale. The same harmonic encodings that are generated by our method can be paired with supervised classifiers to enable crop mapping, since amplitude–phase–mean combinations carry crop-specific signatures. In this way, our proposed method is not only suitable for boundary extraction, but also provides a lightweight, interpretable basis for crop classification.

In this work, we propose a method that integrates harmonic NDVI recolouring with zero-shot segmentation to improve field boundary delineation in fragmented agricultural landscapes. This study addresses a specific question: Does the zero-shot delineation of fields in fragmented farmland improve if we explicitly represent phenology in the input image? We test this on a 5 × 5 km area of interest (AOI) and compare colour spaces and time encodings with object-wise and pixel-wise metrics. We stratify by parcel size to show the impact on parcel sizes. The specific objectives of this research are as follows:

- To evaluate the effectiveness of encoding harmonic NDVI components (mean, amplitude, phase) into cylindrical colour spaces (HSV, HWB, LCH) to improve automatic segmentation of agricultural parcels using SAM.
- To perform quantitative evaluation of segmentation performance with pixel- and object-related metrics, such as Intersection-over-Union (IoU), precision, recall, F1 score, in comparison to manually digitized parcels.
- To investigate how perceptual recolouring affects segmentation accuracy for different parcel configurations, including different sizes, shapes, and landscape contexts.

The remainder of this paper is divided into multiple sections and subsections in which the study area, data sources, and methodological steps are analyzed, including harmonic modelling, cylindrical colour space recolouring, and the use of SAM for segmentation. After this, the experimental results are presented; pixel-based and object-based metrics are reported; and error structures such as fragmentation, over-segmentation, and under-segmentation are examined. In the Section 4, the broader implications of the findings are discussed, including contributions and limitations, and finally, the research is concluded by summarizing the main contributions and outlining directions for future research.

## 2. Materials and Methods

### 2.1. Study Area and PlanetScope Imagery

**Figure 1:**
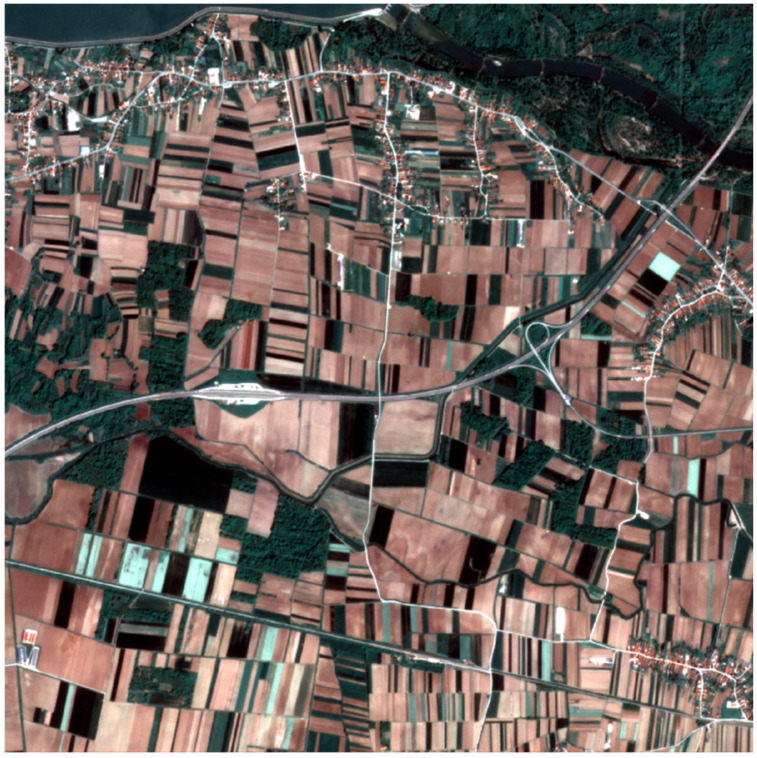
PlanetScope RGB composite of the area of interest, Imagery © 2024 Planet Labs.

The analysis was carried out in a 5 km × 5 km area near Varaždin in northern Croatia. On the PlanetScope scenes (Figure 1), the landscape consists mainly of small agricultural parcels with few hard physical boundaries. Apart from roads and occasional drainage lines, field boundaries are usually not marked by hedges, fences, or rows of trees. Boundaries manifest themselves as subtle shifts in tone, texture, seeding direction, and phenological stages between neighbouring strips.

The scene (Figure 1) shows elongated, rectangular fields. A motorway bisects the area of interest (AOI) and divides it into a northern sector, characterized by smaller agricultural parcels, areas close to the city, the river Drava and its canals, and scattered forests, and a southern sector dominated by larger arable fields with additional forest areas. Dark green patches characterize the forest areas, while pale and reddish-brown tones indicate bare ground. Internal parcel boundaries are generally not expressed as physical features but appear as tonal shifts associated with the phenological stage of the crop. In terms of parcel size, the overwhelming majority of parcels are small; ~70% are smaller than 10,000 m^2^.

The AOI contains the following main classes:

- Agricultural parcels, which consist mainly of small fields, with the median area being ~0.6 ha.
- Grassland and meadows.
- Forests and wooded areas.
- Urban and peri-urban areas concentrated in the northern and south-eastern parts of the AOI.
- Open water bodies and drainage channels concentrated in the northern part of the AOI.

Twenty-two cloud-free SuperDove scenes were acquired for the period from January 2023 to July 2024, covering the AOI at 3 m resolution in eight spectral bands. The average interval between images is 27 days, providing a representative seasonal sample of two full crop cycles. The scenes were obtained through Planet’s Education & Research Program. They were delivered orthorectified and atmospherically corrected in EPSG:32633, and no additional radiometric preprocessing was performed.

### 2.2. Harmonic Analysis

**Figure 2:**
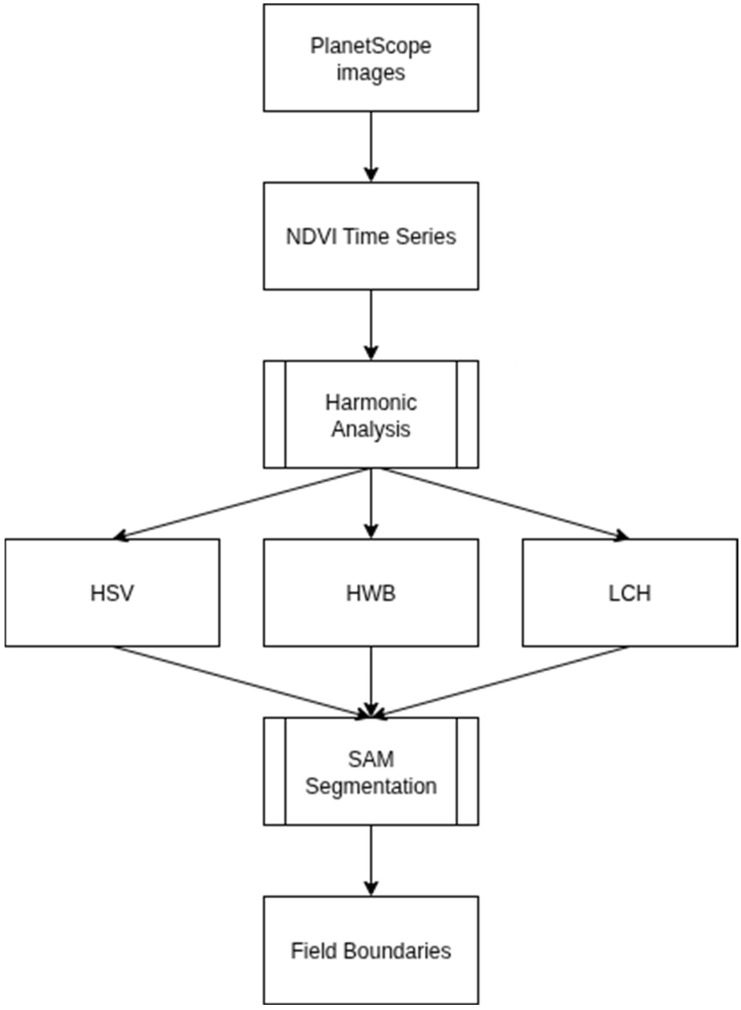
Workflow of the proposed method.

The overall workflow of the proposed method is summarized in Figure 2. Starting with PlanetScope images, NDVI time series are computed and subjected to harmonic analysis to extract the mean, amplitude, and phase. These components are then mapped into cylindrical colour spaces which generate recoloured composites optimized for boundary detection. SAM is subsequently applied to these composites, producing delineated field boundaries for evaluation.

Neighbouring fields tend to have similar reflectivity on any single day, especially near the highest green, making the edges visually indistinct. In contrast, NDVI trajectories differ in phase and amplitude between crops and management regimes. These temporal descriptors are estimated by harmonic decomposition and mapped into cylindrical colour spaces to recolour entire parcels based on phenology, so that neighbouring fields with similar reflectivity have different stable colours in the composite on a single day.

The NDVI was calculated for the entire AOI from 22 cloud-free scenes using the following formula [23]:

(1) $$NDVI=\frac{NIR-RED}{NIR+RED}$$

and processing was performed with Rasterio and NumPy in a single pass over the stacked grids. Acquisition times were encoded as ordinal daily values and stored as a one-dimensional vector aligned to the temporal stack.

Since slow drifts (e.g., background brightness, calibration differences between data) can interfere with a sinusoidal fit, the first step is to remove the pixel-specific linear trend and fit a single harmonic to the residual variability.

The 22-element NDVI series *y*(*t_j_*) of each pixel is regressed on time using ordinary least squares:

(2) $${\hat{y}}_{lin}\left(t\right)=mt+b,$$

The time t is expressed as ordinal daily values, where the unit is days. The residual series

(3) $$r\left(t\right)=y\left(t\right)-{\hat{y}}_{lin}\left(t\right)$$

is transferred to the harmonic stage. In practice, the residuals do not have to be exactly zero-centred due to irregular acquisition times and measurement noise. Therefore, keeping a constant term in the harmonic model catches any small bias that might remain after detrending. This approach is common in time-series modelling, where the primary objective is to isolate seasonal cyclicity rather than to capture multi-year fluctuations. Higher-order polynomials or splines can introduce spurious oscillations in sparsely sampled data [17,18], and risk absorbing variance that should be modelled by the harmonic terms themselves.

The residuals of each pixel are then modelled as a single annual sinusoid with bias:

(4) $$\hat{r}\left(\tau \right)=a_{0}+a_{1}\cos\left(\tau \right)+b_{1}\sin\left(\tau \right),$$

where

(5) $$\tau =\frac{2\pi \times DOY}{365.25},\tau \in \left[0,2\pi \right),\;$$

with τ being the annual angular time (day of the year, converted to radians). The temporal axis was encoded using two formulations. The first one employs an annual radian transformation, in which every day of the year is mapped onto the [0, 2π) interval by assuming a cycle length of 365.25 days. This assumption follows an established practice in Fourier-based vegetation modelling [18], where the tropical year provides a mathematically consistent basis for representing periodic ecological signals.

The mapping sets the fundamental frequency to one cycle per year, so that the fitted coefficients have a direct phenological meaning. The parameters a_0_, a_1_, and b_1_ are estimated by non-linear least squares using the standard Levenberg–Marquardt solver, i.e., they are unweighted, without bounds, and with standard tolerances. The acquisition times are irregular and are passed on directly. No temporal resampling or interpolation is performed. The adjustments are completed without additional failure-handling logic, and the adjusted coefficients (*a*_1_, *b*_1_) are cached in a harmonics.npz file to avoid recalculation.

The calculations were performed using the adjusted coefficients:

(6) $$A=\sqrt{a_{1}^{2}+b_{1}^{2}},A\geq 0,\Phi =atan2\left(b_{1},a_{1}\right),\phi \in \left(-\pi ,\pi \right],$$

The constant term a_0_ represents the residual offset after detrending and is not used in the recolouring. The third colour value uses the empirical NDVI mean over the 22 dates.

Since the harmonic fit uses annual radians τ, ϕ is interpretable phenologically. It encodes the calendar timing in a single year, while A summarizes the seasonal NDVI range. The second approach consists of linear ordinal-day encoding, where the time is expressed simply as the day of the year without forced periodicity. This is due to the fact that different crop types often deviate from strictly annual rhythms due to crop rotations and double harvests, which can create shorter cycles, or make them irregular relative to the astronomical year.

The older harmonic values are also calculated, with the residuals adjusted as follows:

(7) $$\hat{r}\left(t\right)=a_{0}+a_{1}\cos\left(t\right)+b_{1}\sin\left(t\right),\;\;$$

with t in days passed directly to sin and cos. This implicitly sets the period to 2π days, and the resulting *ϕ* is only a relative phase offset, which has been shown to be more effective for visual separation, but cannot be directly interpreted as a day of the year.

### 2.3. Perceptual Recolouring

A is the first harmonic amplitude, and NDVI_mean_ is the empirical mean over the 22 observations. Min-max scaling was selected because cylindrical colour spaces require inputs within [0, 1] to map consistently to hue, saturation, and value. Standardization of the values would generate unbounded values, which would distort the perceptual mapping. To avoid contrast shifts between tiles, AOI width scalers are used, and the following formulae are used in the main experiments:

(8) $$A^{\prime }=\;\frac{A}{\max\left(A\right)},\;\;V^{\prime }=\;\frac{{NDVI}_{mean}+1}{2},\;\;\phi^{\prime }=\phi +\pi ,\;$$

(9) $$A^{\prime }\in \left[0,1\right],\;V^{\prime }\in \left[0,1\right],\;\phi^{\prime }\in \left(0,2\pi \right].$$

**Figure 3:**
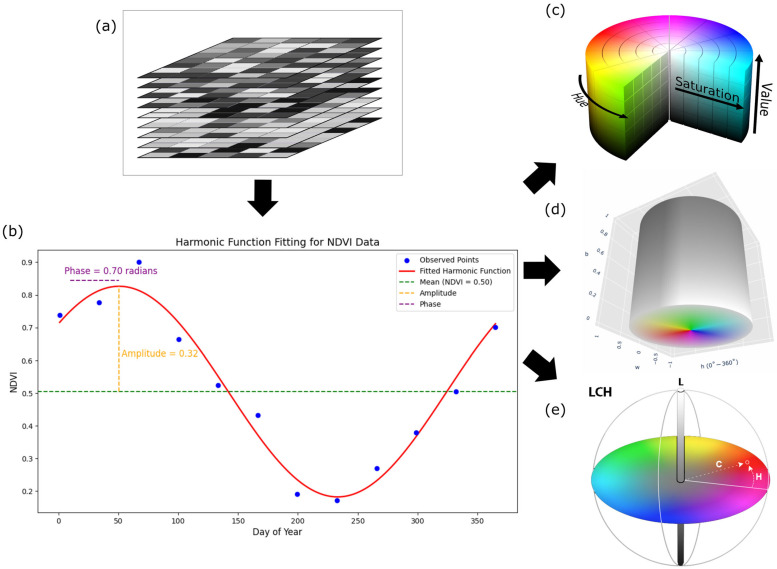
(**a**) Multitemporal NDVI stack; (**b**) harmonic fit per pixel and extraction of parameters; (**c**) mapping to HSV colour space; (**d**) mapping to HWB colour space; (**e**) mapping to LCH colour space.

When recolouring hue and saturation value, hue encodes the phase of the seasonal NDVI cycle so that parcels that peak at different times appear in different colours. As mentioned earlier, hue contains a relative phase offset, which has been shown to be beneficial for visual separation, but does not represent an absolute calendar date. Saturation increases with the seasonal range. Fields with stronger NDVI variations are more vivid, while weak seasonal and perennial vegetation appears pastel-coloured. The value is directly related to the average greenness of the time series, so greener parcels appear brighter, even if their timing is similar. Figure 3 illustrates the entire workflow of recolouring from a multitemporal NDVI stack to different cylindrical colour spaces. Figure 3a shows the input in the form of a stack of NDVI images acquired on different days. By stacking the NDVI images, a time series is obtained for each pixel. Figure 3b shows the observed NDVI values of a pixel over the course of a year. A single harmonic model is fitted to the sample, from which three values are derived, the amplitude, which quantifies the seasonal range, the phase, which encodes the timing of the seasonal peak, and the mean, which is the empirical average of the NDVI over the data for a pixel. Figure 3c shows the mapping of the above values to the HSV colour space to produce a single three-band image suitable for segmentation. The phase (timing) is mapped to the hue, the amplitude to the saturation, and the value is determined by the empirical mean NDVI values. The assignment of the phase to the hue separates the neighbouring parcels by the timing, while the saturation and the value modulate the contrast by the seasonal range and the mean value.

In the case of the Hue–Whiteness–Blackness colour space, the hue again separates the parcels according to phenological time. The brightness is directly proportional to the amplitude, i.e., strongly seasonal fields appear bright, and areas with low seasonality appear darker. The whiteness comes from the raw NDVI mean, which desaturates greener parcels more. The result is a palette with a clear temporal separation, a brightness controlled by seasonality, and a saturation softened by the average green. Figure 3d illustrates the mapping of the above parameters into the HWB colour space to produce a single three-band image.

In the Luminance–Chroma–Hue colour space, the luminance represents the mean green intensity, the chroma represents the seasonal strength, and the hue angle represents the timing. As the chroma is extended to the entire area, parcels with strong seasonality are given a high colour purity, while weakly seasonal areas collapse towards grey. To avoid clipping outside the sRGB boundaries, chroma values were adaptively scaled to the maximum valid range, ensuring that hue and luminance relationships were preserved without distortion. Figure 3e illustrates the assignment of the three factors to the LCH colour space. The phase corresponds to the hue; chroma is the normalized amplitude; and the lightness is the mean NDVI. The LCH triplet is then rendered using the standard LCH–LAB–RGB conversion, in which the timing, seasonal range, and baseline greenness are encoded as separate visual channels.

### 2.4. Segmentation with Segment Anything Model

We applied SAM independently to three recoloured composites—HSV, HWB, and LCH—and generated a segmentation mask with the native resolution of 3 m at each iteration. The composites were passed to SAM as Float32 RGB arrays, with no additional normalization or contrast stretching beyond the recolouring mappings mentioned above. No land cover masks were applied, and inference was performed on the entire AOI.

Tiles of 512 × 512 px were used for inference. Tile generation, error handling, multi-scale cropping, and tile merging was performed automatically by the Segment Geospatial Wrapper that surrounds SAM’s Automatic Mask Generator. The following parameters were defined according to [24,25], and coarsely adjusted within reasonable ranges to produce satisfying results:

(10) crop_n_layers=2, crop_overlap_ratio=0.35, crop_n_points_downscale_factor=1, 

(11) points_per_side=32, pred_iou_thresh=0.75, stability_score_thresh=0.80 .

These values are closely related to [25], and our settings fall within the typical ranges used with SAM. The parameters were not cherry-picked for performance but selected to reasonably balance coverage and precision in fragmented fields.

SAM ViT-H was used, with the weights sam_vit_h_4b8939.pth. The merging of masks across overlapping tiles was performed automatically by SAM’s internal non-maximum suppression (NMS) and deduplication logic. The resulting outputs were not merged and each of the colour spaces was processed separately. This resulted in six independent prediction sets. The instance masks were then converted to vector polygons. No holes were filled; no morphological operations were performed; and no edge smoothing algorithms were applied. To assess whether tiling and internal NMS introduced edge artifacts, a seam-interior analysis was conducted. Tile boundaries were buffered by 16 pixels and rasterized to create seam and interior zones. Ground truth and predictions were rasterized at the native 3 m resolution, and pixel-wise IoU, precision, recall and F1 score were computed separately in the seam and interior zones to quantify whether segmentation accuracy was systematically degraded near tile edges.

### 2.5. Validation and Accuracy Metrics

The reference layer consists of hand-digitized parcel polygons for the AOI. The ground truth contains 1304 polygons, covering ~13 km^2^. Both the ground truth and the image data have the same spatial reference and were automatically aligned. The ground truth was digitized by the authors for this AOI and serves exclusively as a reference for the segmentation experiments conducted in this article.

**Figure 4:**
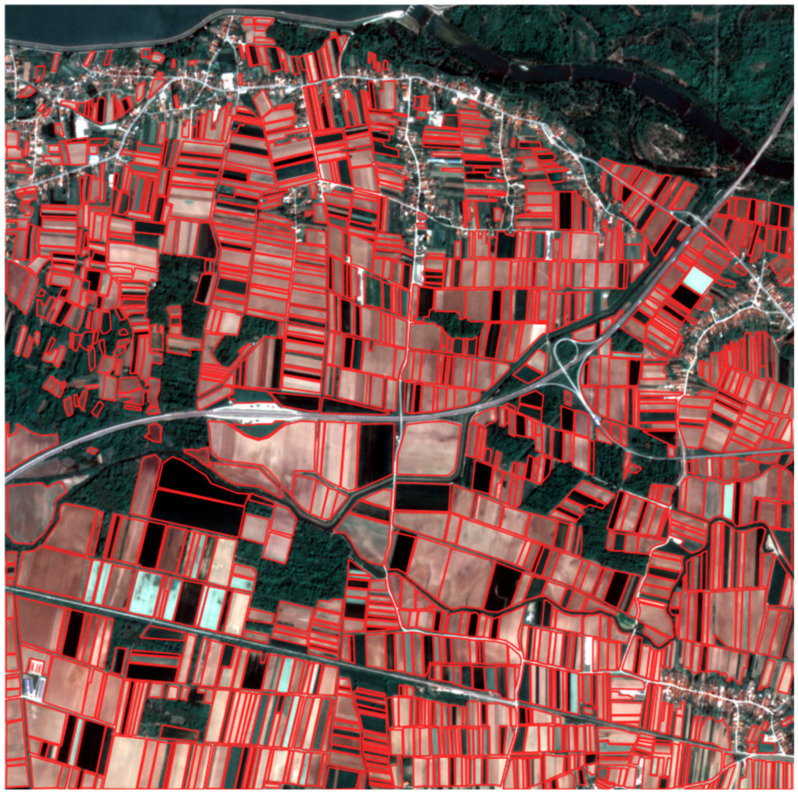
The ground truth parcels and the RGB composite, Imagery © 2024 Planet Labs.

Figure 4 shows the ground truth overlaid with the RGB composite of the scene. All accuracy metrics are calculated within the AOI boundaries, and the derived geometries are clipped to the AOI extent to avoid edge artefacts. The same evaluation pipeline and the same threshold values were used for all three colour spaces. The object-related metrics are calculated in vector space, and the pixel-related metrics were calculated by rasterising the ground truth and predictions to the native resolution of 3 m.

Prior to evaluation, the predictions were filtered by removing polygons with an area of less than 30 m^2^. In addition, a preselection was made using the intersection-over-union (IoU) method, where for each predicted polygon the best IoU is calculated for each parcel of ground truth and only the predictions with an IoU > 0.50 are retained. In this way, the predictions that plausibly match agricultural parcels are selected so that only these are included in the evaluation. For future iterations, the IoU-based hand selection step can be changed by training a simple classifier (e.g., Random Forest, Support Vector Machines…) on ~100 labelled parcels.

With respect to the object-based metrics, the retained predictions and ground truth are used to match the predictions and ground truth. By iterating over the ground truth polygons in order of occurrence, a spatial index for the prediction is queried to obtain candidate polygons whose bounding box intersects the current ground truth parcel. In cases where a candidate does not yet match, the IoU value is evaluated and the polygon with the highest value is selected. If this IoU value is at least 0.50, the ground truth and prediction pairing is registered as a match and both objects are marked as used. Each ground truth parcel can be matched with a maximum of one prediction, and each prediction can participate in one match. After matching the predictions and the ground truth, true-positive predictions (TPs) are predictions that have been assigned to a prediction with an IoU value of more than 0.50. False-positive predictions (FPs) are predictions that have not been assigned to a ground truth, and false-negative predictions (FNs) are ground truth polygons that have not been assigned to a prediction.

These numbers were used to calculate the following metrics:

(12) $$Precision=\frac{TP}{TP+FP},Recall=\frac{TP}{TP+FN},F1=\frac{2*Precision*Recall}{Precision+Recall}$$

To quantify where each colour space representation helps or fails, the object-based metrics were also categorized by the area of ground-truth parcel area. The parcels were divided into three categories:

- Parcels with an area of less than 5000 m^2^.
- Parcels with an area between 5000 m^2^ and 20,000 m^2^.
- Parcels with an area of more than 20,000 m^2^.

Within each of these categories, the following were counted:

- TP—ground truth parcels in the category that were matched to exactly one prediction with IoU > 0.50.
- FN—ground truth parcels in the category with no matching predictions.
- FP—predictions that did not match any ground truth parcels.

These counts were used to calculate recall, precision, F1, and the mean IoU for each category. True negatives (TNs) are not shown, as the evaluation is object-based and ground-truth anchored, and the TN would represent the background, which is irrelevant to the calculated metrics.

Pixel-wise metrics are also calculated by rasterizing both the ground truth and each prediction level. The vector polygons were burnt as binary masks (parcel = 1, background = 0), and in the case where the prediction polygons overlapped, the raster array stored the union. The following counts are calculated on this grid:

- TP—cells where the ground truth mask and the prediction mask both equal 1, correctly covered parcel area.
- FP—cells where the ground truth mask is zero, and the prediction mask is one, polygons in the non-agricultural area.
- FN—cells where the ground truth mask is equal to one, and the prediction mask is equal to zero, missing parcel areas.

As mentioned above, TN is not counted as it represents the background, which is irrelevant for the calculated metrics. Using the previously mentioned counts, the following metrics are calculated: precision, recall, F1, and IoU:

(13) $$IoU=\frac{TP}{TP+FP+FN}$$

IoU measures how well a prediction matches the ground truth.

To quantify fragmentation, the number of predicted polygons associated with each ground truth parcel is calculated. A ground truth polygon is considered fragmented if it is matched to more than one prediction. The fragmentation metrics are stratified by parcel area.

In addition to fragmentation counts, object-level geometric error metrics are also calculated. These include Global Over-Classification Error (GOC), Global Under-Classification Error (GUC), and Global Total Classification Error (GTC) [12]. *S_i_* denotes the i-th predicted parcel, and *O_i_* indicates the ground truth parcel with which *S_i_* has the largest intersection. For each prediction, the Over-Classification error is defined per [12] as

(14) $$OC\left(S_{i}\right)=1-\frac{area\left(S_{i}\;\cap \;O_{i}\right)}{area\left(O_{i}\right)},\;$$

and the under-classification error as

(15) $$UC\left(S_{i}\right)=1-\frac{area\left(S_{i}\;\cap \;O_{i}\right)}{area\left(S_{i}\right)},\;$$

the total classification error as per [12] is

(16) $$TC\left(S_{i}\right)=\sqrt{\frac{OC{\left(S_{i}\right)}^{2}+UC{\left(S_{i}\right)}^{2}}{2}}$$

Global error measures were obtained as area-weighted means across all predictions to quantify segmentation accuracy in terms of spatial overreach, omission, and overall geometric consistency [12]:

(17) $$GOC=\;\sum_{i}^{n}w_{i}OC\left(S_{i}\right),\;GUC=\sum_{i}^{n}w_{i}OC\left(S_{i}\right),\;GTC=\sum_{i}^{n}w_{i}TC\left(S_{i}\right)$$

(18) $$w_{i}=\frac{area\left(S_{i}\right)}{\sum_{k}^{n}area\left(S_{k}\right)}$$

Statistical significance testing was conducted to assess whether the observed performance differences between colour-space variants were systematic rather than due to random variation. Because each parcel was evaluated under all six colour-space transformations, the scores form a repeated-measure design. A nonparametric Friedman test was applied as an omnibus test on parcel-level IoU and F1 scores within each size category. The Friedman test evaluates whether at least one method differs in rank distribution across parcels [26]. In the cases where the test was significant, a pairwise Wilcoxon signed-rank test was performed between all method pairs, with Holm correction to control the family-wise error rate [26,27]. This combination provides a robust alternative to paired *t*-tests, which assume normally distributed differences and are less suited to bounded and skewed performance metrics, such as IoU and F1 [26,27].

To quantify potential biases introduced by manual annotation, the area of interest was re-digitized independently a second time by another operator. The two datasets were compared parcel by parcel to measure inter-annotator consistency. For every matched pair of parcels, the IoU and GTC were calculated, and precision, recall, and F1 score were calculated by rasterizing each parcel pair to the native 3 m PlanetScope resolution. The matched pairs were categorized into three groups, just as before.

## 3. Results

### 3.1. Application of Perceptual Recolouring

#### 3.1.1. Ordinal Day Fit

**Figure 5:**
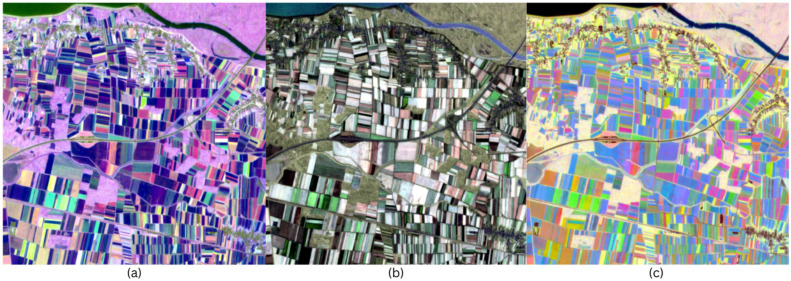
(**a**) HSV recoloured image; (**b**) HWB recoloured image; (**c**) LCH recoloured image.

Legacy recolouring assigns phase (timing), seasonal amplitude, and mean NDVI, all estimated from a single harmonic fit to ordinal days, to perceptual channels. Figure 5 shows the resulting recoloured images. Figure 5a depicts the recoloured HSV composite, image. It leads to strong hue separation between neighbouring fields with different phenologies, and small parcels that are similar in one-date images are now given different colours. Low-amplitude areas desaturate towards grey tones, and high-amplitude crops appear in strongly saturated colours. The mean NDVI value controls the brightness, so that the river and built-up areas are dark. The change in hue tone can also be seen throughout the scene. Fields with slightly different phenologies appear in hues that are close to each other. Figure 5b shows the newly coloured HWB composite. It appears less vividly coloured than the other colour spaces. Persistently green parcels appear whitewashed, strongly seasonal crops appear brighter, and there is less chromatic blurring. The interior of the parcels is evenly coloured and the contrast is carried by the hue and lightness. Figure 5c shows the LCH recoloured composite image, which is characterized by a perceptibly uniform lightness. In the areas where the seasonal amplitude is strong, the colour intensity is high, and the parcels are vividly coloured, while the lower-amplitude areas appear rather muted. The colour hues are similarly time-dependent, but the separation is by chroma, rather than saturation or value.

#### 3.1.2. Annual-Radian Fit

**Figure 6:**
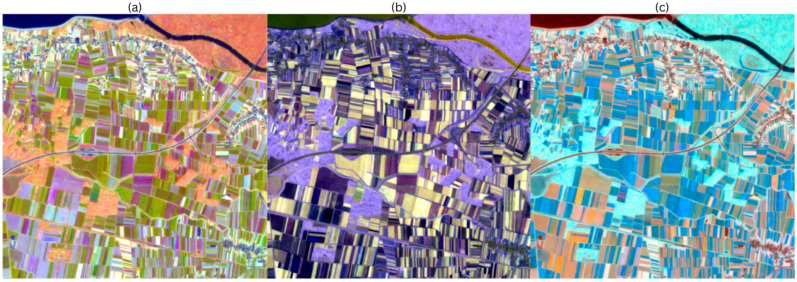
(**a**) HSV annual-radian recoloured image; (**b**) HWB annual-radian recoloured image; (**c**) LCH annual-radian recoloured image.

With seasonal-radian time (τ), the phase becomes directly interpretable as calendrical timing, so that fields that peak around the same part of the year coincide and form similar hues. Figure 6 depicts the recoloured images in the annual radian variant. Figure 6a represents the HSV annual-radian recoloured image, and the scene still shows good parcel coherence, but the hue between adjacent parcels is narrower, and many adjacent parcels fall into similar colours because their peaks are close to each other calendrically. Parcels with low amplitude appear grey. Parcels with high amplitude are vividly saturated. Water and built-up areas remain dark, and persistent green areas remain brightly coloured. Figure 6b depicts the HWB annual radian scene, recoloured. Persistent green parcels appear whiter, and strongly seasonal crops appear brighter in colour. Hues are grouped by calendar year, so that the colour differences are due to changes in brightness in addition to slight shifts in hue. The interior of the parcels is uniform, and urban areas and water bodies appear dark. Figure 6c is the scene recoloured by LCH in the annual-radian recoloured scene, again resulting in a perceptibly uniform lightness. Chroma scales with amplitude, so that strong seasonal variations appear more vivid, while low-amplitude areas appear pastel-coloured. Parcels that peak in the same season rotate to closely spaced hue angles, so separation depends on the combination of timing and range.

### 3.2. Segmentation Outputs

#### 3.2.1. Ordinal Day Fit

**Figure 7:**
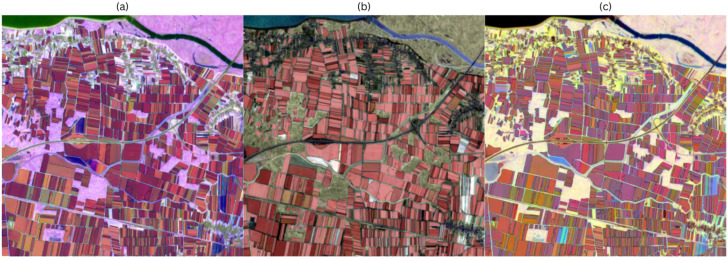
(**a**) HSV segmentation output; (**b**) HWB segmentation output; (**c**) LCH segmentation output.

Figure 7 illustrates the zero-shot segmentation on the recoloured composite images with ordinal days, with the masks superimposed on the input image. Figure 7a depicts the result for the HSV composite image. Coverage is dense; most fields receive at least one mask with high overlap, and the interiors are mostly clean and free of holes. Non-stable boundaries are limited and typically occur where neighbouring parcels have a similar mean greenness and amplitude but differ slightly in phase. Figure 7b shows the output for the HWB composite. The predicted masks match well with elongated, rectangular field geometries. However, some small fields with low amplitude are overlooked or merged with the neighbouring parcels. Figure 7c shows the predicted masks for the LCH composite. The interior of the parcels is stable, and holes are rare, but there are some merging masks in the areas where the amplitude is modest, and the timing is similar in the neighbouring parcels. The boundaries still follow visible tonal shifts, but the separation is weaker than in the HSV or HWB colour spaces.

#### 3.2.2. Annual-Radian Fit

**Figure 8:**
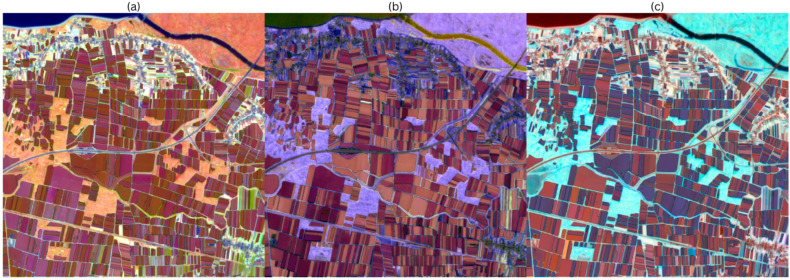
(**a**) HSV annual-radian segmentation output; (**b**) HWB annual-radian segmentation output; (**c**) LCH annual-radian segmentation output.

Figure 8 depicts the segmentation results at zero-shot segmentation output on the yearly recoloured composites. Figure 8a is the output for the composite with HSV annual radian colours. The interior of the parcels remains coherent and mostly free of holes, but the colour contrast between parcels is weaker than in ordinal day matching, so SAM tends to merge more masks. Small stripes are still recognized, but the duplicates cluster around larger patches. Figure 8b shows the output on the annually recoloured HWB image. The predicted masks match well with the long field edges, and fragmentation is less frequent. Whiteness and inverse blackness continue to provide a strong brightness structure, so that medium and large parcels are reliably detected, while smaller parcels are occasionally missed. Figure 8c depicts the output on the LCH annual radian image. As phase is mapped to hue, and amplitude to chroma; separation is based on subtle differences; and masks are less reliably predicted than in previous colour spaces.

### 3.3. Validation Metrics

**Figure 9:**
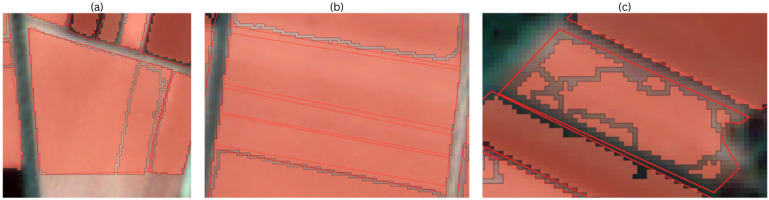
Examples of the main segmentation error types compared to the ground truth. The red borders represent the ground truth data, and the light red polygons represent the predictions. (**a**) Over-segmentation: a single ground truth parcel is split into multiple predicted polygons, visible as parallel red polygons inside one field; (**b**) under-segmentation: several neighbouring parcels are merged into a larger predicted mask; (**c**) fragmentation: a single ground truth parcel is broken into numerous, small, irregular predictions scattered inside the black boundary.

The performance of the method is reported at the prediction levels under a ground-truth anchored matching protocol (IoU > 0.50). The object-based scores show whether the system returned a geometry per true parcel; the tables categorized by size show how the behaviour changes with parcel area; and the pixel-based scores quantify the area match on a common 3 m grid. Three recurring errors, identifiable for each colour space, have a significant impact on the object-based metrics:

- Over-segmentation—an event where the model suggests multiple masks for a single parcel. A case of over-segmentation is shown in Figure 9a. Over-segmentation lowers precision while recall remains high.
- Under-segmentation—a case where a single predicted polygon spans multiple parcels of ground truth. As a result, recall decreases while the precision remains stable. An example of sub-segmentation is shown in Figure 9b.
- Fragmentation—an event where the model provides many small parts for a ground truth parcel. If at least one part has an IoU > 0.50, we obtain a TP and many FPs, resulting in lower precision. An example of fragmentation can be found in Figure 9c.

#### 3.3.1. Object-Wise Metrics

Table 1 depicts the object-related metrics, with the columns representing the individual metrics, precision, recall, and F1, and the rows representing the individual colour spaces, both for the annual radian and for the ordinal daily adjustment. HWB provides the best overall balance, with the highest F1 value and precision, but also has the fewest predicted polygons, resulting in a less fragmented output. HSV achieves the highest recall, but at the cost of lower precision and the generation of more additional polygons. LCH has a similar recall to HSV, but even lower precision, and the largest number of predictions among the non-annual radian segmentations. Switching from ordinal days to annual radians reduces the F1 score for all colour spaces, indicating weaker separability with this approach for this AOI.

#### 3.3.2. Object-Wise Metrics Categorized by Area

To see how the metrics change from tiny strips to large parcels, the ground truth parcels are grouped into three areas. For each of these areas, the values for precision, recall F1, and mean Intersection-over-Union (mIoU) are calculated. Mean Intersection-over-Union represents the mean value of Intersection-over-Union for all matching pairs.

Table 2 depicts the object-related results for the smallest fields, i.e., the fields with an area of less than 5000 m^2^. Ordinal day fit HSV achieved both the highest recall and F1 value and the second best mIoU value. HWB performed worse for F1 but delivered the highest precision. Switching to annual radians reduced the metrics in all cases, except for recall in the case of HWB.

Table 3 shows the property-specific results for the medium-sized fields, with an area between 5000 m^2^ and 20,000 m^2^. HWB provides the best overall balance with the highest F1 score. HSV ranks second, with the highest recall, mIoU, but lower precision. mIoU also increases to ~0.78–0.81, and the change from ordinal days to annual radians lowers all metrics.

Table 4 shows the object-related metrics for parcels larger than 20,000 m^2^. Recall and mIoU are the highest overall, as large fields are found reliably, but precision decreases due to over-segmentation, under-segmentation, and fragmentation. This explains the discrepancy with the overall trend, while pixel-wise IoU increases with parcel size because boundary errors are proportionally smaller. Object-wise precision can still fail when SAM returns multiple masks for the same parcel. HWB still has the highest F1 score and precision, and HSV still has the highest recall and mIoU, while LCH still trails behind. Switching to annual radians lower the scores for all colour spaces once more.

#### 3.3.3. Pixel-Wise Metrics

Table 5 shows the pixel-wise metrics. All variants achieve a high score, confirming that the interior of the parcels is well covered in all colour spaces. HSV has the highest F1 value and mIoU, while HWB has the highest precision and recall. All colour spaces achieve high values for the pixel-wise metrics. The switch to annual radians leaves the pixel-wise metrics almost unchanged.

#### 3.3.4. Tile Boundary Errors

Table 6 depicts the comparison between seam zones, which are 16-pixel buffers around tile borders, and interior regions for the six colour space variants. While all metrics were lower at seams, the drops were limited. IoU decreased by 0.017–0.046, the F1 score was lowered by 0.011–0.029. The consistency across colour spaces shows that the error is systematic, but minor. Notably, the relative performance hierarchy remained unchanged, confirming that tiling-related artifacts did not distort the main findings.

#### 3.3.5. Fragmentation Metrics

Table 7, Table 8 and Table 9 report the fragmentation ratios, defined as the number of predicted parcels relative to the number of ground truth parcels, grouped by parcel size. The ratio represents how many parcels are predicted on average for each ground truth parcel.

Table 7 depicts the results for parcels whose areas are less than 5000 m^2^, and the ratios range between 1.15 and 1.41, which indicates that the fragmentation is systematic even at the smallest scale. HWB produces the least amount of fragmentation, while the annual radian versions of the HSV and HWB colour space produce the most.

Table 8 reports the metrics for medium parcels, whose area is between 5000 m^2^ and 20,000 m^2^, and it is noticeable that fragmentation became more pronounced, with ratios climbing up to 1.93 for the annual radian HSV colour space. LCH and the annual radian variants show particularly high fragmentation, approaching two predicted polygons for every ground truth parcel.

Table 9 reports the results for large parcels whose area is greater than 20,000 m^2^, and the ratio increases sharply, ranging from 1.92 for HWB to 3.86 for annual radian LCH. This demonstrates that large, homogenous fields are consistently over-segmented, and typically split into two or more predicted polygons. Amongst other colour spaces, HWB shows the lowest fragmentation, while LCH and annual radian variants fragment the most.

The results show that fragmentation is a systematic bias of SAM delineations, and its severity increases with parcel sizes. HWB consistently produces the fewest fragments, followed by HSV, while LCH and annual radian variant consistently produce the most.

#### 3.3.6. Over-Segmentation and Under-Segmentation Metrics

Table 10, Table 11 and Table 12 report the Global Over-Classification Error (GOC), Global Under-Classification Error (GUC), and their combined measure, the Global Total Classification Error (GTC), grouped by parcel area.

The results for small parcels with areas of less than 5000 m^2^ are reported in Table 10. Both GOC and GUC are low, with the values in range of 0.017–0.024, and the errors are minimal because when predictions exist for small parcels, they tend to fit tightly. The combined GTC is consistently low across all variants, confirming that omission and spillover are rare in this size class.

Medium-sized parcel metrics, whose area is between 5000 m^2^ and 20,000 m^2^, are reported in Table 11, and the errors are the highest in this size group. GOC values reach 0.106 for the annual radian LCH variant, and GUC values reach up to 0.087 for the annual radian HSV variant. GTC ranges from 0.098 up to 0.118, which indicates that medium-sized parcels are the most prone to both spillover and omission. This aligns with the fragmentation results, as medium parcels are complex enough to produce multiple predicted fragments, but not large enough for SAM to maintain stable interiors.

The results for large parcels, whose area is greater than 20,000 m^2^ are reported in Table 12. Their GOC values range between 0.049 for the HWB colour space and 0.095 for the annual radian LCH variant, while the GUC values are lower. The combined GTC was 0.044–0.086, lower than for medium parcels. These results show that large parcels are dominated by over-segmentation, with relatively little omission of their interiors, which is consistent with the observation of fields splitting into multiple fragments.

Across all parcel sizes, HWB consistently produces the lowest or near-lowest GOC and GUC values, which confirms its robustness in limiting both spillover and omission. HSV performs competitively, especially for small parcels, while LCH and annual radian variants produce the highest errors.

#### 3.3.7. Digitization Uncertainty

Table 13 reports the inter-annotator agreement metrics between the two independent manual digitisations, grouped by parcel area. The agreement was consistently high across all three categories, with F1 scores exceeding 0.94, and IoU values being above 0.89 for the smallest parcels. As the parcel size increased, the agreements improved, reaching a maximum IoU of 0.968 and an F1 score of 0.983 for parcels larger than 20,000 m^2^. The GTC showed the same trend, decreasing from 0.053 in the smallest category to 0.016 in the largest category. Precision and recall values were both very high in all cases.

#### 3.3.8. Statistical Significance Testing

Table 14 presents the Friedman omnibus test results across parcel size categories. For clarity, only one set of values is reported, since the test statistics and *p*-values are identical for IoU and F1 in each size bin. The results confirm highly significant differences between different colour spaces in all categories, with the strongest effect appearing in the category of small parcels (<5000 m^2^).

Pairwise Wilcoxon signed-rank tests with Holm correction were applied to identify which colour space variants differed significantly. Selected results are reported in Table 15 (F1 score), and Table 16 (IoU), with the full comparisons provided in Appendix A. Table A1 reports the pairwise Wilcoxon signed-rank tests for the F1 score, and Table A2 reports those for the IoU. Several patterns emerge consistently, the first of them being that HSV significantly outperforms LCH (F1: +0.009, IoU: +0.015; both *p* < 0.001), while the differences between HWB and LCH were not significant (*p* > 0.6). And although HWB achieved the numerically highest F1 score in Table 1, its margin over HSV is very small despite reaching statistical significance, which confirms that the observed advantage is real, but negligible in practical terms. The annual radian variants did not yield systematic gains, with the HSV and LCH annual radian variants performing significantly worse than their base counterparts, while annual radian HWB variant is statistically indistinguishable from the ordinal day HWB.

## 4. Discussion

### 4.1. Comparison with Previous Works

The recolouring of the multitemporal NDVI into cylindrical colour spaces and the segmentation of the resulting image with a zero-shot segmenter leads to reliable field delineation without retraining. The approach generates consistently parcel-coherent colour fields for SAM. The three colour spaces emphasize different aspects of the time series, leading to systematic precision–recall trade-offs. This method produces per-pixel amplitude and phase patches that are robust to slow drifts and can be used directly for remapping the perceived colour space. In practice, HWB tends to produce results with the lowest number of fragments, while HSV favours coverage, and LCH is somewhere in between.

Classical edge detectors are based on local gradients, region-based and integrated methods merge/split by spectral similarity. This method differs in that it generates contrast from time, and not just space, which is useful for boundaries. A compact harmonic model represents the temporal progression (phase), seasonal bandwidth (amplitude), and mean green colouration as separable visual channels. Conceptually, this behaves like a regional cue in which parcel-coherent colours emerge, which SAM uses as an implicit region during the mask proposal. Compared to heavily supervised methods, this method is lightweight and interpretable, it requires no labelled data and no fine-tuning, and it exploits seasonality, which other methods typically ignore.

Building on this zero-shot foundation, more recent customized SAM variants such as [28,29] show clear ways to improve performance. A complementary supervised path is [30], which reports performance improvements with a strong generalization of zero-shot generalization across resolutions and regions. While our pipeline is label-free and interpretable, ref. [30] suggests a backbone that could be paired with recoloured input. Reference [29] can be combined with our recolourings without the need for retraining. This provides a cost-effective way to sharpen images while preserving parcel-coherent interiors. The segmentation head of [30], YoloV11, can be used as a topology repair model.

Beyond delineation, the harmonic NDVI encodings used in this research also carry crop-specific information, phase reflects growth timing, amplitude captures seasonal intensity, and the mean tracks baseline greenness. These descriptors are discriminative for crop classification, suggesting that our method can be extended by pairing the recoloured composites with supervised classifiers. In such a setup, the segmentation step would provide parcel boundaries, while the harmonic features embedded in colour channels would serve as inputs for crop identification, linking field geometry and crop type in a unified workflow.

### 4.2. Error Analysis and Methodological Uncertaintie

In all colour spaces, the dominant errors are over-segmentation, fragmentation, and under-segmentation. These errors explain the high recall and high correspondence IoU, where the boundaries are usually well placed on the best mask, but whose precision is reduced by additional polygons. By slightly fine-tuning SAM, the above errors can be reduced [28,29,30]. In particular, ref. [28] couples a DeeplabV3+ prompter with SAM and fine-tunes the decoder. A hybrid approach could be tested directly by keeping the recoloured composites as inputs and training a prompter on a modest labelled subset to direct the prompters to the interior of the parcels. Similarly, ref. [29] shows that improving the inputs with principal component analysis (PCA), high-frequency decomposition, and guided filtering can make the SAM embeddings more marginal, which could reduce the under-segmentation in densely packed strips.

HSV makes phase differences clearly visible via hue and strongly pushes seasonal crops towards saturated colours, maximizing detection but also promoting over-segmentation on large, uniform parcels. HWB encodes the mean NDVI value as whiteness and the amplitude as inverse blackness, dampening mottling and suppressing duplicates, improving object-level precision. The perceptual uniformity of LCH often results in cleaner, more uniform parcel fills, as well as smoother hue transitions across gradual timing gradients. However, using the full chroma range can result in some colours hitting the sRGB limit and the converter clipping the out-of-range values. For this reason, chroma was scaled iteratively during rendering. This adjustment preserved the relative relationships between hue, luminance, and chroma, ensuring that seasonality strength remained interpretable while avoiding artifacts.

Using ordinal days instead of mapping the day of the year annual radians changes the representation of the phase. The mapping of the annual cycle has the advantage that it can be directly interpreted phenologically, since the phase corresponds to a calendar angle tied to a 365.25-day tropical year [18]. In our area of interest, fitting ordinal days resulted in a stronger contrast between parcels and a higher SAM separability than the annual radian model. A possible explanation for this is that the relaxation of the annual period constraint allows the sinusoid to absorb sub-annual management signals, such as double cropping, which exaggerates inter-parcel differences and facilitates segmentation. This suggests that alternative encodings such as the ordinal day encoding may offer greater agroclimatic sensitivity in fragmented agricultural systems.

It is important to note that in natural colour imagery, the brightness differences typically stem from crop type and growth stage, while in our method, the brightness is directly proportional to the NDVI amplitude. This means that strongly seasonal fields appear brighter and weakly seasonal areas appear darker. This mapping reduces the influence of short-lived spectral changes, such as flowering, and instead emphasizes the strength of seasonal vegetation dynamics. As a result of this, the brightness of the recoloured composites should be interpreted as a phenological indicator rather than a visual analogue of crop reflectance properties.

### 4.3. Input Data and Environmental Uncertainties

Some strengths of this method are that it requires no training, is quick to apply, and is interpretable. It is index-independent and sensor-independent. Within these limits, the cost advantage is also convincing. By using 22 cloud-free images and no labelling outside the evaluation set, the pipeline delivers strong results with little technical effort. The improvements are straightforward and incremental, and do not compromise the simplicity of the method.

Limitations remain. The results were derived from a single area in Croatia, a single sensor family, and a single harmonic seasonal model, so generalization to other landscapes requires further testing. The ground truth digitized by hand also tends to contain biases at the boundaries. However, these factors do not undermine the observations; rather, they motivate broader validation across different landscapes, agricultural contexts, and additional sensors such as Sentinel-2, as well as a series of robustness checks.

In addition, multitemporal datasets are inherently sensitive to atmospheric disturbances, residual cloud contamination, and imperfect radiometric normalization, all of which may reduce the stability of harmonic fitting and the consistency of phase and amplitude estimates. Small misregistrations can produce artificial boundary signals, particularly in fragmented landscapes where parcels are only a few pixels wide. The assumption of a single dominant seasonal harmonic does not capture more complex or irregular crop cycles, such as double cropping or intercropping, which could lead to mischaracterization of parcel-level dynamics.

Within these limits, the pipeline shows promising results, but careful attention to preprocessing quality, sensor consistency, and agro-ecological context is critical to ensure robust transferability to larger and more diverse agricultural regions.

## 5. Conclusions

This work proposes a method that recolours harmonic NDVI time series into cylindrical colour spaces to expose the parcel-level structure to a zero-shot segmenter. In a 5 × 5 km landscape without strong physical boundaries, this approach consistently provides a coherent appearance of the parcels and enables effective delineation without additional training or fine-tuning.

Among the tested colour spaces, HSV prioritizes coverage, encodes phase as hue, increases boundary contrast between fields with asynchronous phenology, and delivers the highest recall and best pixel-wise F1 score and IoU. HWB produces the cleanest masks. Amplitude luminance reduces fragmentation and achieves the highest precision and best overall object-based F1 score, especially for medium and large parcels. LCH typically lies between the other two colour spaces.

In summary, the proposed method of harmonic NDVI recolouring combined with SAM segmentation provides a solid, transparent basis for parcel delineation. It is quick to deploy, easy to explain, and adaptable to different operational objectives. This approach is well positioned to deliver value today, while providing a clear path for future improvements. Beyond delineation, the harmonic descriptors (phase, amplitude, and mean) embedded in the recoloured composites also hold strong discriminative properties for crop classification, suggesting that our method can be extended to jointly delineate fields and identify crop types in a single, scalable workflow.

## Figures and Tables

**Table 1:** Object-wise segmentation metrics.

Colour	Precision	Recall	F1
HSV	0.509	0.750	0.607
HWB	0.555	0.677	0.610
LCH	0.448	0.744	0.559
HSV—annual radians	0.393	0.644	0.488
HWB—annual radians	0.415	0.698	0.520
LCH—annual radians	0.381	0.655	0.482

**Table 2:** Object-wise metrics for parcels < 5000 m^2^.

Colour	Precision	Recall	F1	mIoU
HSV	0.563	0.669	0.612	0.742
HWB	0.599	0.545	0.571	0.744
LCH	0.561	0.665	0.609	0.723
HSV—annual radians	0.440	0.511	0.473	0.726
HWB—annual radians	0.512	0.612	0.557	0.731
LCH—annual radians	0.458	0.523	0.488	0.699

**Table 3:** Object-wise metrics for parcels 5000 m^2^–20,000 m^2^.

Colour	Precision	Recall	F1	mIoU
HSV	0.503	0.789	0.614	0.810
HWB	0.559	0.743	0.638	0.805
LCH	0.435	0.783	0.560	0.785
HSV—annual radians	0.402	0.712	0.514	0.782
HWB—annual radians	0.417	0.739	0.533	0.793
LCH—annual radians	0.410	0.727	0.524	0.771

**Table 4:** Object-wise metrics for parcels larger than 20,000 m^2^.

Colour	Precision	Recall	F1	mIoU
HSV	0.412	0.864	0.558	0.864
HWB	0.455	0.856	0.594	0.858
LCH	0.301	0.848	0.444	0.825
HSV—annual radians	0.284	0.816	0.421	0.814
HWB—annual radians	0.261	0.824	0.397	0.815
LCH—annual radians	0.213	0.800	0.337	0.803

**Table 5:** Pixel-wise metrics.

Colour	Precision	Recall	F1	mIoU
HSV	0.949	0.852	0.898	0.815
HWB	0.956	0.858	0.887	0.798
LCH	0.944	0.830	0.883	0.791
HSV—annual radians	0.933	0.843	0.886	0.795
HWB—annual radians	0.945	0.853	0.897	0.813
LCH—annual radians	0.942	0.819	0.876	0.780

**Table 6:** Tile boundary errors.

Colour	ΔIoU	ΔPrecision	ΔRecall	ΔF1
HSV	0.040	0.016	0.031	0.024
HWB	0.046	0.030	0.041	0.029
LCH	0.027	0.070	0.017	0.017
HSV—annual radians	0.029	0.012	0.023	0.018
HWB—annual radians	0.030	0.015	0.021	0.018
LCH—annual radians	0.017	0.012	0.011	0.011

**Table 7:** Fragmentation metrics for parcels < 5000 m^2^.

Colour	Number of Ground Truth Parcels	Number of Predicted Polygons	Ratio
HSV	505	690	1.37
HWB	505	580	1.15
LCH	505	690	1.37
HSV—annual radians	505	712	1.41
HWB—annual radians	505	700	1.39
LCH—annual radians	505	683	1.35

**Table 8:** Fragmentation metrics for parcels 5000 m^2^–20,000 m^2^.

Colour	Number of Ground Truth Parcels	Number of Predicted Polygons	Ratio
HSV	674	1127	1.67
HWB	674	975	1.45
LCH	674	1284	1.91
HSV—annual radians	674	1299	1.93
HWB—annual radians	674	1285	1.91
LCH—annual radians	674	1296	1.92

**Table 9:** Fragmentation metrics for parcels > 20,000 m^2^.

Colour	Number of Ground Truth Parcels	Number of Predicted Polygons	Ratio
HSV	125	274	2.19
HWB	125	240	1.92
LCH	125	362	2.90
HSV—annual radians	125	377	3.02
HWB—annual radians	125	401	3.21
LCH—annual radians	125	483	3.86

**Table 10:** Over-segmentation and under-segmentation metrics for parcels < 5000 m^2^.

Colour	GOC	GUC	GTC
HSV	0.021	0.014	0.021
HWB	0.017	0.021	0.023
LCH	0.024	0.014	0.024
HSV—annual radians	0.020	0.020	0.024
HWB—annual radians	0.021	0.020	0.024
LCH—annual radians	0.022	0.018	0.025

**Table 11:** Over-segmentation and under-segmentation metrics for parcels 5000 m^2^–20,000 m^2^.

Colour	GOC	GUC	GTC
HSV	0.077	0.077	0.099
HWB	0.074	0.083	0.098
LCH	0.102	0.065	0.107
HSV—annual radians	0.100	0.087	0.118
HWB—annual radians	0.100	0.074	0.107
LCH—annual radians	0.106	0.075	0.112

**Table 12:** Over-segmentation and under-segmentation metrics for parcels > 20,000 m^2^.

Colour	GOC	GUC	GTC
HSV	0.054	0.028	0.052
HWB	0.049	0.019	0.044
LCH	0.077	0.021	0.065
HSV—annual radians	0.082	0.043	0.082
HWB—annual radians	0.082	0.025	0.070
LCH—annual radians	0.095	0.037	0.086

**Table 13:** Digitization uncertainty metrics, grouped by parcel area.

Area	Precision	Recall	F1	IoU	GTC
<5000 m^2^	0.930	0.963	0.944	0.898	0.053
5000 m^2^–20,000 m^2^	0.953	0.979	0.964	0.934	0.034
>20,000 m^2^	0.980	0.988	0.983	0.968	0.016

**Table 14:** Results of the Friedman test.

Area	Number of Parcels	Q	*p*-Value
<5000 m^2^	505	129.262	3.41 × 10^−26^
5000 m^2^–20,000 m^2^	674	105.520	3.62 × 10^−21^
>20,000 m^2^	125	39.399	1.97 × 10^−7^

**Table 15:** Results of pairwise Wilcoxon signed-rank tests for F1 scores across colour-space variants.

Variant A	Variant B	Median F1 Difference	Lower 95% CI ^1^	Upper 95% CI ^1^	Adjusted *p*-Value ^2^
HSV	HWB	0.0028	0.0000	0.0062	5.96 × 10^−5^
HSV	LCH	0.0092	0.0068	0.0117	1.63 × 10^−10^
HSV—Annual Radians	HSV	−0.0222	−0.0266	−0.0164	1.46 × 10^−23^
LCH	LCH—Annual Radians	−0.0217	−0.0290	−0.0161	1.13 × 10^−19^

^1^ Confidence Interval; ^2^ Holm-adjusted *p*-value.

**Table 16:** Results of pairwise Wilcoxon signed-rank tests for IoU scores across colour-space variants.

Variant A	Variant B	Median IoU Difference	Lower 95% CI ^1^	Upper 95% CI ^1^	Adjusted *p*-Value ^2^
HSV	HWB	0.0044	0.0000	0.0084	5.64 × 10^−5^
HSV	LCH	0.0146	0.0107	0.0187	2.49 × 10^−11^
HSV—Annual Radians	HSV	−0.0328	−0.0401	−0.0246	1.69 × 10^−25^
LCH	LCH—Annual Radians	−0.0320	−0.0392	−0.0234	8.85 × 10^−20^

^1^ Confidence Interval; ^2^ Holm-adjusted *p*-value.

## Data Availability

Restrictions apply to the availability of these data. The PlanetScope imagery analyzed in this study was obtained under a research license from Planet Labs PBC and cannot be redistributed. Derived data (figures) are included in the article.

## Abbreviations

The following abbreviations are used in this manuscript:

RGB	Red Green Blue.
NDVI	Normalized Difference Vegetation Index.
SAM	Segment Anything Model.
AOI	Area of Interest.
HSV	Hue–Saturation–Value.
HWB	Hue–Whiteness–Blackness.
LCH	Luminance–Chroma–Hue.
IoU	Intersection-over-Union.
TP	True Positive.
FP	False Positive.
FN	False Negative.
TN	True Negative.
NMS	Non-Maximum Suppression.
PCA	Principal Component Analysis.
GEE	Google Earth Engine.
GLCM	Gray Level Co-occurrence Matrix.
GOC	Global Over-Classification Error.
GUC	Global Under-Classification Error.
GTC	Global Total Classification Error.

## Appendix A

To complement the main text, this appendix reports the complete results of the pairwise Wilcoxon signed-rank tests comparing segmentation performance across all colour-space variants. Both F1 scores and IoU scores were tested following a Friedman test that confirmed statistically significant differences among methods.

Table A1 and Table A2 show the pairwise comparisons for F1 and IoU, respectively. For each pair of methods, the table reports the median difference in scores, the 95% bootstrap Confidence Interval (CI), and the Holm-adjusted *p*-value correcting for multiple testing. A positive median difference indicates that the first listed method performed better than the second listed method. HSV and LCH annual radian variants differ significantly from their base counterparts, often showing systematic performance gaps. HSV and LCH comparisons consistently favour HSV, with significant positive differences in both F1 and IoU. HWB and its annual radian variant are statistically indistinguishable in both metrics, despite numerical differences, indicating their relative stability.

**Table A1:** Pairwise Wilcoxon signed-rank test results for F1 scores across colour-space variants.

Variant A	Variant B	Median F1 Difference	Lower 95% CI ^1^	Upper 95% CI ^1^	Adjusted *p*-Value ^2^
HSV—Annual Radians	LCH—Annual Radians	0.0043	0.0014	0.0083	2.47 × 10^−2^
HSV—Annual Radians	HWB—Annual Radians	−0.0082	−0.0107	−0.0054	2.03 × 10^−10^
HSV—Annual Radians	HSV	−0.0222	−0.0266	−0.0164	1.46 × 10^−23^
HSV—Annual Radians	LCH	−0.0114	−0.0173	−0.0051	2.83 × 10^−11^
HSV—Annual Radians	HWB	−0.0107	−0.0145	−0.0055	1.56 × 10^−5^
LCH—Annual Radians	HWB—Annual Radians	−0.0173	−0.0235	−0.0125	2.73 × 10^−14^
LCH—Annual Radians	HSV	−0.0310	−0.0399	−0.0246	3.48 × 10^−29^
LCH—Annual Radians	LCH	−0.0217	−0.0290	−0.0161	1.13 × 10^−19^
LCH—Annual Radians	HWB	−0.0191	−0.0270	−0.0132	3.74 × 10^−6^
HWB—Annual Radians	HSV	−0.0069	−0.0130	−0.0037	1.94 × 10^−4^
HWB—Annual Radians	LCH	0.0000	−0.0043	0.0048	8.87 × 10^−1^
HWB—Annual Radians	HWB	0.0000	−0.0008	0.0032	6.00 × 10^−1^
HSV	LCH	0.0092	0.0068	0.0117	1.63 × 10^−10^
HSV	HWB	0.0028	0.0000	0.0062	5.96 × 10^−5^
LCH	HWB	−0.0065	−0.0095	−0.0013	6.17 × 10^−1^

^1^ Confidence Interval; ^2^ Holm-adjusted *p*-value.

**Table A2:** Pairwise Wilcoxon signed-rank test results for IoU scores across colour-space variants.

Variant A	Variant B	Median IoU Difference	Lower 95% CI ^1^	Upper 95% CI ^1^	Adjusted *p*-Value ^2^
HSV—Annual Radians	LCH—Annual Radians	0.0067	0.0014	0.0117	2.52 × 10^−2^
HSV—Annual Radians	HWB—Annual Radians	−0.0115	−0.0158	−0.0073	1.20 × 10^−10^
HSV—Annual Radians	HSV	−0.0328	−0.0401	−0.0246	1.69 × 10^−25^
HSV—Annual Radians	LCH	−0.0157	−0.0253	−0.0071	3.05 × 10^−11^
HSV—Annual Radians	HWB	−0.0154	−0.0208	−0.0084	2.02 × 10^−6^
LCH—Annual Radians	HWB—Annual Radians	−0.0247	−0.0326	−0.0155	7.48 × 10^−15^
LCH—Annual Radians	HSV	−0.0493	−0.0574	−0.0382	1.51 × 10^−31^
LCH—Annual Radians	LCH	−0.0320	−0.0392	−0.0234	8.85 × 10^−20^
LCH—Annual Radians	HWB	−0.0278	−0.0364	−0.0209	2.39 × 10^−7^
HWB—Annual Radians	HSV	−0.0111	−0.0197	−0.0047	4.14 × 10^−5^
HWB—Annual Radians	LCH	0.0000	−0.0054	0.0060	7.97 × 10^−1^
HWB—Annual Radians	HWB	0.0000	−0.0010	0.0057	1.00 × 10^0^
HSV	LCH	0.0146	0.0107	0.0187	2.49 × 10^−11^
HSV	HWB	0.0044	0.0000	0.0084	5.64 × 10^−5^
LCH	HWB	−0.0091	−0.0154	−0.0019	8.04 × 10^−1^

^1^ Confidence Interval; ^2^ Holm-adjusted *p*-value.
